# Identifying differential interactions of potentially traumatic experiences with genetic risk for posttraumatic stress disorder

**DOI:** 10.1080/20008066.2026.2709319

**Published:** 2026-09-16

**Authors:** Jacob Knyspel, Saakshi Kakar, Anna Carnegie, Gursharan Kalsi, Laura Meldrum, Iona Smith, Shannon Bristow, Molly R. Davies, Chérie Armour, NIHR BioResource, Thalia C. Eley, Gerome Breen, Chloe C.Y. Wong, Jonathan R.I. Coleman

**Affiliations:** a Social, Genetic & Developmental Psychiatry Centre, Institute of Psychiatry, Psychology, and Neuroscience, King's College London, London, UK; b National Institute for Health and Care Research Biomedical Research Centre, King's College London and South London and Maudsley National Health Service Trust, London, UK; c Stress, Trauma, and Related Conditions (STARC) Research Centre, School of Psychology, Queen's University Belfast, Belfast, UK; d Cambridge University Hospitals NHS Foundation Trust, Cambridge Biomedical Campus, Cambridge, UK

**Keywords:** PTSD, trauma, genetics, polygenic risk scores, gene-environment interaction, TEPT, trauma, genética, puntajes de riesgo poligénico, interacción gen-ambiente

## Abstract

**Background:** Posttraumatic stress disorder (PTSD) occurs following potentially traumatic experiences, but not everyone who experiences trauma develops PTSD. Genetic factors play an important role in shaping how individuals respond to trauma, reflecting gene-environment interaction. An open and important question in PTSD genetics research is the extent to which gene-environment interactions vary across specific traumas.

**Objective:** We aimed to compare gene-environment interaction effects across a broad range of self-reported potentially traumatic experiences across the lifespan.

**Method:** We analysed existing data from two large UK cohorts: the UK Biobank and GLAD-EDGI-COPING (N = 144,702). PTSD symptoms were assessed using the six-item abbreviated PTSD Checklist. We examined eleven self-reported trauma exposures, including five childhood traumas and six adulthood traumas. We conducted gene-environment interaction analyses for each trauma at three levels of genetic specificity: polygenic risk scores, specific genes, and specific genetic variants.

**Results:** Childhood traumas showed stronger associations with PTSD symptoms and greater gene-environment interactions than adulthood traumas on average. Childhood emotional and physical neglect also demonstrated greater gene-environment interactions than childhood abuse. Interaction patterns varied across genes and variants, with several leading PTSD-associated genes (e.g. *ANAPC, FAM120A, SGCD*) having particularly strong interactions with certain traumas. Several genes (*DTX4, PSMD12, TYW3, ZNF660*) demonstrated consistently stronger interactions across all childhood or all adulthood traumas. Differences in gene-environment interactions across traumas were not explained by variation in exposure rates, associations with PTSD symptoms, or gene-environment correlations.

**Conclusions:** Our findings are consistent with differential gene-environment interactions on PTSD across potentially traumatic experiences, with those in childhood exhibiting the strongest interactions. To account for the moderating role of trauma type on genetic associations, genetic research on PTSD should incorporate detailed trauma exposure information, which may improve PTSD risk prediction and support more personalised prevention approaches.

## HIGHLIGHTS

- Genetic factors may influence how people respond to potentially traumatic experiences, which shapes individual risk for PTSD.
- We found that genetic factors interacted more strongly with childhood traumas than adulthood traumas, and particularly with childhood emotional/physical neglect.
- We also found that many genes varied in the traumas they interacted most strongly with.
- Our results highlight the importance of accommodating trauma exposure information in PTSD genetics research.

## Abstract (es)

**Antecedentes:** El trastorno de estrés postraumático (TEPT) puede desarrollarse tras la exposición a experiencias potencialmente traumáticas; sin embargo, no todas las personas expuestas a un trauma desarrollan TEPT. Los factores genéticos desempeñan un papel importante en la forma en que los individuos responden al trauma, lo que refleja la existencia de una interacción entre los genes y el ambiente. Una pregunta abierta y de gran relevancia en la investigación genética del TEPT es hasta qué punto las interacciones entre los genes y el ambiente varían según tipos específicos de trauma.

**Objetivo:** Nuestro objetivo fue comparar los efectos de las interacciones entre los genes y el ambiente en un amplio espectro de experiencias potencialmente traumáticas autorreportadas a lo largo de la vida.

**Método:** Analizamos datos existentes de dos grandes cohortes del Reino Unido: el UK Biobank y GLAD-EDGI-COPING (N = 144.702). Los síntomas de TEPT se evaluaron mediante la Lista de Verificación de TEPT versión abreviada de seis ítems. Examinamos once exposiciones a traumas autorreportadas, incluidas cinco correspondientes a traumas en la infancia y seis a traumas en la adultez. Realizamos análisis de interacción entre los genes y el ambiente para cada tipo de trauma en tres niveles de especificidad genética: puntajes de riesgo poligénico, genes específicos y variantes genéticas específicas.

**Resultados:** En promedio, los traumas ocurridos durante la infancia mostraron asociaciones más fuertes con los síntomas de TEPT y mayores interacciones entre los genes y el ambiente que los traumas ocurridos durante la adultez. Asimismo, la negligencia emocional y física en la infancia mostró interacciones entre los genes y el ambiente de mayor magnitud que el abuso infantil. Los patrones de interacción variaron entre genes y variantes genéticas, y varios de los principales genes asociados con el TEPT (p. ej., *ANAPC*, *FAM120A*, *SGCD*) presentaron interacciones particularmente intensas con determinados tipos de trauma. Varios genes (*DTX4*, *PSMD12*, *TYW3*, *ZNF660*) mostraron interacciones consistentemente más fuertes en todos los traumas de la infancia o en todos los traumas de la adultez. Las diferencias en las interacciones entre los genes y el ambiente según el tipo de trauma no pudieron explicarse por variaciones en las tasas de exposición, en la magnitud de las asociaciones con los síntomas de TEPT ni en las correlaciones entre los genes y el ambiente.

**Conclusiones:** Nuestros hallazgos son consistentes con la existencia de interacciones diferenciales entre los genes y el ambiente en el TEPT según el tipo de experiencia potencialmente traumática, siendo las experiencias ocurridas durante la infancia las que exhiben las interacciones más intensas. Para considerar el papel moderador del tipo de trauma sobre las asociaciones genéticas, la investigación genética del TEPT debería incorporar información detallada sobre la exposición a traumas, lo que podría mejorar la predicción del riesgo de TEPT y favorecer el desarrollo de estrategias de prevención más personalizadas.

## 1. Introduction

Posttraumatic stress disorder (PTSD) is a common mental health condition that emerges following exposure to potentially traumatic experiences (Yehuda et al., 2015). Many traumas are known to contribute to PTSD, examples of which (e.g. sexual violence, war exposure) are listed in the DSM-5 and ICD-11 to aid clinicians (American Psychiatric Association, 2022; World Health Organization, 2024). A diagnosis of PTSD is dependent on these environmental events, but not everyone who experiences trauma develops PTSD, and among those who do, some are more severely affected than others (Galatzer-Levy et al., 2018). Risk of PTSD following trauma is shaped by environmental factors (Tortella-Feliu et al., 2019) and genetic variation (Duncan et al., 2018). Estimates of the heritability of PTSD from twin studies have ranged from 24% to 72% (Duncan et al., 2018), indicating a substantial proportion of individual variance in PTSD risk corresponds to genetic variance. The largest genome-wide association study (GWAS) of PTSD to date, which included data from over 150,000 people with PTSD, identified 95 loci significantly associated with the condition (Nievergelt et al., 2024). Downstream analyses identified 43 genes with a potentially causal effect. Genomic research has provided valuable insight into the neurobiology of PTSD, implicating genes that influence stress response, threat processing, emotion regulation, and the immune system (Dalvie et al., 2021; Polimanti & Wendt, 2021).

Given the necessary role of trauma in PTSD, the influence of genetics must be viewed through the lens of gene-environment interaction (Koenen et al., 2008; Seah et al., 2025). Gene-environment interaction occurs when the effect of an environmental factor differs among people based on their genetics (Virolainen et al., 2022). As a general example, skin cancer risk from sun exposure is greater among individuals with fairer skin (Garbe et al., 2024), which is shaped by genetics. For PTSD, the key gene-environment interaction of interest is whether the risk conferred by a given trauma is influenced by the genetics of the people who experience it. Many candidate gene studies have examined interactions between trauma exposure and variants linked to individual genes, primarily related to stress response (Seah et al., 2025). However, these studies have typically been restricted to small sample sizes, and candidate gene studies have generally failed to replicate (Border et al., 2019; Duncan & Keller, 2011). A recent study employed a two-stage approach in which GWAS was performed and associated variants were then analysed for gene-environment interactions (Marchese et al., 2022). The authors identified three significant variant-trauma interaction effects, suggesting a genome-wide approach based on exploratory analyses, rather than assumed biological theories, is worthwhile.

An open question in PTSD research is to what extent gene-environment interactions vary across specific traumas. Traumas vary in the risk conferred for PTSD, with sexual violence typically conferring the greatest risk (Kessler et al., 2017; Schincariol et al., 2024). Many studies have found that traumas can be associated with different symptom presentations (Forbes et al., 2012; Kelley et al., 2009; Smith et al., 2016), which could partly be explained by variation in how traumas interact with genetics, although this remains under-explored. Candidate gene studies have typically focused on single traumas, ranging from childhood abuse to combat exposure (Seah et al., 2025), without comparing traumas systematically. Some large genomic studies of PTSD have used cohorts that might be assumed to have particular trauma exposures, such as military cohorts (Huckins et al., 2020; Stein et al., 2021). However, using trauma-specific cohorts is more conceptually and statistically limited than directly measuring and comparing traumas (Marchese & Huckins, 2023), which is essential for quantifying differences in interactions across traumas and identifying potentially trauma-specific interactions.

In this study, we explored variation in gene-environment interaction effects on PTSD, comparing eleven potentially traumatic experiences measured across the lifespan. We aimed to build upon prior research by quantifying differences in interaction effects among a broad range of traumas and incorporating three levels of genetic specificity: polygenic risk scores (PRS), genes, and single nucleotide polymorphisms (SNPs). We used data from two cohorts in the United Kingdom: the UK Biobank (UKB; N = 108,919) (Davis et al., 2020; Sudlow et al., 2015), a population-scale resource drawn from volunteers from the general population; and GLAD-EDGI-COPING (GEC; *N* = 35,783) (Davies et al., 2019; Monssen et al., 2024; Young et al., 2023), which includes individuals with lifetime experience of mental health problems.

## 2. Method

### 2.1. Cohorts and measures

We analysed existing data from two cohorts in the United Kingdom: the UK Biobank (Davis et al., 2020; Sudlow et al., 2015) (UKB; *N* = 108,919) and the National Institute for Health and Care Research BioResource GLAD-EDGI-COPING studies (GEC), which include the Genetic Links to Anxiety and Depression study (Davies et al., 2019) (GLAD; *N* = 21,813), the Eating Disorders Genetics Initiative UK (Monssen et al., 2024) (EDGI; *N* = 3,026), and participants in the COVID-19 Psychiatry and Neurological Genetics study (Young et al., 2023) (COPING; *N* = 10,944). We included only participants of European genetic ancestries and excluded genetically related participants based on a shared relatedness greater than third degree. GEC participants also in UKB were removed from the GEC data. The proportion of female participants was higher in GEC (73%) than UKB (56%), while the mean age of participants was higher in UKB (56.1, SD = 8.0) than GEC (44.2, SD = 18.2).

In GEC, PTSD symptoms were self-reported using the six-item abbreviated PTSD checklist (Lang & Stein, 2005) (PCL-6). Participants were presented with six items about core PTSD symptoms (e.g. ‘Feeling very upset when something reminded you of a stressful experience’; see Supplementary Method for items) and asked to report how much they have been bothered by each within the past month, with options ranging from 1 (‘Not at all’) to 5 (‘Extremely’). Total PCL-6 scores ranged from 6 to 30. In UKB, PTSD symptoms were self-reported using the PCL-6 with a few alterations. One of the PCL-6 questions (difficulty concentrating) was not presented to participants. We therefore used responses to a comparable item from the PHQ-9 (‘Recent trouble concentrating on things’). Options for this item ranged from 1 (‘Not at all’) to 4 (‘Nearly every day’), meaning that total scores ranged from 6 to 29. Two PCL-6 items were gated such that unless participants responded >1 to any of the 3 other items they were not shown these items (Supplementary Method). If participants had missing data for these gated items, they were imputed as 1 (‘Not at all’).

Childhood trauma exposure was self-reported using the Childhood Trauma Screener (Glaesmer et al., 2013; Grabe et al., 2012). Participants were presented with brief descriptions of five traumas (emotional abuse, emotional neglect, physical abuse, physical neglect, sexual abuse) and asked how true each description was to their childhood, with response options ranging from 0 (‘Never true’) to 4 (‘Very often true’). Binary cutoffs for trauma exposure were based on previous research (Glaesmer et al., 2013) and varied by trauma (see Supplementary Method for items and cutoffs). Adulthood trauma exposure was self-reported using a brief checklist in which participants were asked to report whether they had ever experienced six traumas (serious accident, serious illness, sexual assault, violent crime, war exposure, witnessing death). Response options included 0 (‘Never’), 1 (‘Yes, but not in the last 12 months’), and 2 (‘Yes, in the last 12 months’). Participants were considered trauma exposed if they responded 1 or 2 for a given trauma.

We used imputed SNP data to form the basis of our polygenic risk score, gene, and SNP analyses. For quality control and imputation information, see Supplementary Method. We also refer to published descriptions for each cohort (Bycroft et al., 2018; Davies et al., 2019; Monssen et al., 2024; Young et al., 2023). We included SNPs with a minor allele frequency ≥0.01 and imputation quality (INFO) score ≥0.8.

### 2.2. PRS-trauma interaction analyses

We calculated PRS for PTSD and major depressive disorder (for sensitivity analysis) using PRScs (Huckins et al., 2019) with default parameters (Supplementary Method) and the summary statistics from the most recent PGC GWAS of each condition excluding the UK Biobank (Adams et al., 2025; Nievergelt et al., 2024). Within each cohort, we estimated additive PRS-trauma interaction effects using 13 separate linear regression models (11 traumas plus any childhood and any adulthood trauma) with PCL-6 scores as the outcome. These models included genotyping batch and 10 genomic principal components as covariates, as well as PRS-covariate and trauma-covariate interactions. We performed fixed-effect meta-analyses on the PRS-trauma interaction effects from each cohort using the ‘meta’ *R* package (Schwarzer et al., 2015). To test whether meta-analysed interaction effects differed significantly across traumas, we used Z-score comparisons, calculated as the difference between effects relative to their standard errors:

$$Z=\frac{\beta_{1}-\beta_{2}}{\sqrt{SE_{1}^{2}+SE_{2}^{2}}}$$

We calculated Spearman correlations between the meta-analysed PRS-trauma interaction effects and (1) the exposure rate of each trauma; (2) the point-biserial correlation between each trauma and PCL-6 scores; (3) the point-biserial correlation between each trauma and PTSD PRS; and (4) the degree of overlap of each trauma with other traumas. The degree of overlap was calculated as the sum of the phi coefficients between a trauma and the other traumas. Point-biserial correlations were calculated using the ‘ltm’ *R* package (Rizopoulos, 2006). As sensitivity analyses, we reran these steps using PRS for major depressive disorder (MDD), which is highly genetically correlated with PTSD (Adams et al., 2025; Nievergelt et al., 2024), and reran the childhood trauma models with these traumas as ordinal variables (0-4) instead of binary variables. To assess the influence of PRS-trauma correlations on PRS-trauma interactions, we used structural equation models (via the ‘lavaan’ *R* package [Rosseel, 2012]) that were equivalent to the primary analyses except also included a freely estimated path from PRS → trauma. We compared PRS-trauma interaction estimates to those from alternative models in which this path was constrained to zero. This would allow us to calculate attenuation in interaction effects when explicitly modelling gene-environment correlation. For additional information about the structural equation models, see Supplementary Method.

### 2.3. Gene-trauma interaction analyses

We used PRSet (Choi et al., 2023; Choi & O’Reilly, 2019) to calculate risk scores for 387 of the 415 protein coding genes taken from Supplementary Table 9 of the most recent PGC PTSD GWAS (Nievergelt et al., 2024). PRSet is an extension of the PRS software PRSice-2 that allows users to calculate scores within specific genomic regions of interest. We calculated scores for each gene by inputting their respective base position ranges taken from the same supplementary table (in build hg19 for UKB and build hg38 for GEC; see Supplementary Table 10 for list of genes). We used the same summary statistics as the PRS analyses. See Supplementary Method for PRSet parameters used. PRSet could not produce a risk score for the remaining 28 of the 415 genes, and so these were excluded (see Supplementary Method for list).

We estimated additive gene-trauma interaction effects using separate linear regression models with PCL-6 scores as outcome. This totalled 5,031 models (13 traumas across 387 genes). These models included the same covariates as the PRS-trauma models. We again performed fixed-effect meta-analyses on the gene-trauma interaction effects and used Z-score comparisons to test differences between traumas. We performed omnibus testing on each gene to assess whether its mean interaction effect with childhood traumas differed significantly from its mean interaction effect with adulthood traumas. To do this, we used the ‘systemfit’ *R* package (Henningsen & Hamann, 2007) to rerun the 11 specific trauma models as a system of simultaneous equations and directly test the null hypothesis that the mean effects are equivalent. We meta-analysed the omnibus test *p*-values across cohorts using Fisher’s method. We conducted gene enrichment analyses using Fisher’s exact tests to compare genes with and without (1) significant PCL-6 score associations, (2) significant interaction effects, and (3) significant omnibus tests, against a total of 63 gene ontology classes (see Supplementary Table 11 for list). These classes were selected as they were shared by at least 3 of the 387 genes examined. A positive result in an enrichment analysis would indicate that the significant genes (e.g. genes with significant interaction effects) had a higher probability of belonging to a given class than non-significant genes.

### 2.4. SNP-trauma interaction analyses

We extracted allele scores for the 95 lead SNPs from the most recent PGC PTSD GWAS (Nievergelt et al., 2024) using the ‘–recode A’ PLINK2 (Chang et al., 2015) command. We again estimated additive SNP-trauma interaction effects using separate linear regression models (1,235 in total) with PCL-6 scores as outcome. These models included the same covariates as the PRS-trauma and gene-trauma models. We again performed fixed-effect meta-analyses on the SNP-trauma interaction effects and calculated Z-score comparisons among traumas.

## 3. Results

### 3.1. Trauma exposure

Across both cohorts, 65% of participants reported exposure to at least one trauma (Table 1). Sexual abuse was the most common childhood trauma (16%), while sexual assault and violent attacks were the most common adulthood traumas (20%). Compared to UKB, GEC had a higher rate of trauma exposure in childhood (51% vs 27%) and adulthood (60% vs 51%). Childhood emotional neglect had the greatest difference across cohorts (31% vs 6%). Phi coefficients among traumas ranged from 0.00 to 0.72 in UKB (median = 0.07; Supplementary Table 1) and −0.01–0.67 in GEC (median = 0.12; Supplementary Table 2). Thus, exposure to any trauma was almost always associated with increased risk of exposure to every other trauma.

**Table 1.** Trauma exposure rates (%) by cohort and sex.

	GLAD-EDGI-COPING			UK Biobank		
Trauma	Overall (*N* = 35,783)	Male (*N* = 9,333)	Female (*N* = 25,514)	Overall (*N* = 108,919)	Male (*N* = 47,915)	Female (*N* = 61,004)
*Childhood trauma*						
Emotional abuse	31.5	19.5	34.9	9.6	6.9	11.7
Emotional neglect	31.2	27.2	32.7	6.1	5.6	6.5
Physical abuse	15.3	13.6	16.0	8.3	8.2	8.3
Physical neglect	3.6	3.6	3.6	3.0	3.0	3.1
Sexual abuse	15.7	8.9	18.2	16.2	15.2	17.1
Any childhood trauma	50.7	41.6	53.5	27.2	25.5	28.5
*Adulthood trauma*						
Serious accident	11.6	15.9	10.2	10.0	13.5	7.1
Serious illness	9.8	11.9	9.3	16.2	17.7	15.1
Sexual assault	36.2	13.9	44.3	15.0	7.7	20.7
Violent attack	22.8	35.5	18.7	19.3	25.0	14.9
War exposure	2.4	5.5	1.4	3.7	6.3	1.7
Witnessing death	13.8	20.0	11.7	13.7	19.4	9.2
Any adulthood trauma	60.2	60.2	60.2	51.1	56.3	47.0
Any childhood or adulthood trauma	74.1	72.2	74.7	62.3	65.8	59.5
*Number of traumas*						
0	26.7	28.4	26.1	37.7	34.2	40.5
1	23.8	27.6	22.5	30.9	31.9	30.1
2	18.0	17.1	18.2	16.9	18.3	15.7
3	12.8	11.4	13.2	7.8	8.7	7.1
4	8.4	7.1	8.9	3.7	3.8	3.6
5 or more	10.3	8.4	11.1	3.0	3.1	3.0

Note: Self-reported sex data were missing for 936 participants in GLAD-EDGI-COPING.

### 3.2. PTSD symptoms

Across both cohorts, the mean PCL-6 score was 11.1 (SD_pooled_ = 4.4). Applying the typical cutoff of 14, 16% of participants screened positively for PTSD. Scores were greater in GEC (mean = 13.8, SD = 6.5) than UKB (mean = 8.0, SD = 3.1), translating to 45% of GEC participants and 7% of UKB participants screening positively. Across both cohorts, childhood emotional abuse was associated with the greatest mean PCL-6 score (Supplementary Table 3). All traumas were positively associated with PTSD symptom scores to some extent, with point-biserial correlations ranging from 0.01 to 0.39 in GEC (median = 0.22) and 0.03 to 0.27 in UKB (median = 0.12; Supplementary Table 4).

### 3.3. PRS-trauma interactions

**Figure 1.**
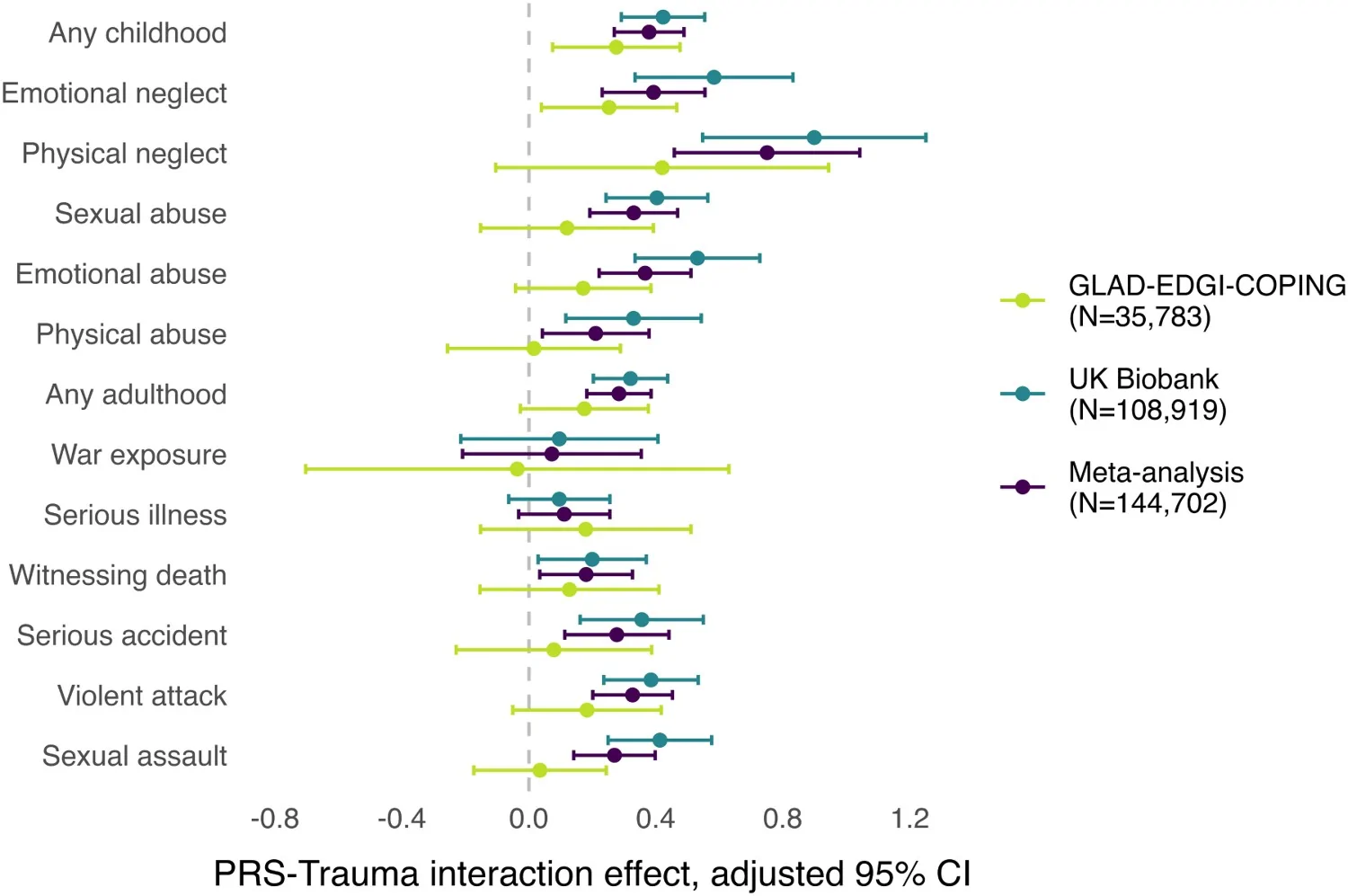
PTSD PRS-trauma interaction effects from both cohorts and fixed-effect meta-analysis. Note: CI: Confidence interval; PRS: Polygenic risk score. Confidence intervals were Bonferroni adjusted to account for 39 interaction effects (13 traumas across 3 cohorts). A positive interaction effect indicates the effect of a trauma on PTSD increases as genetic risk for the condition also increases.

**Figure 2.**
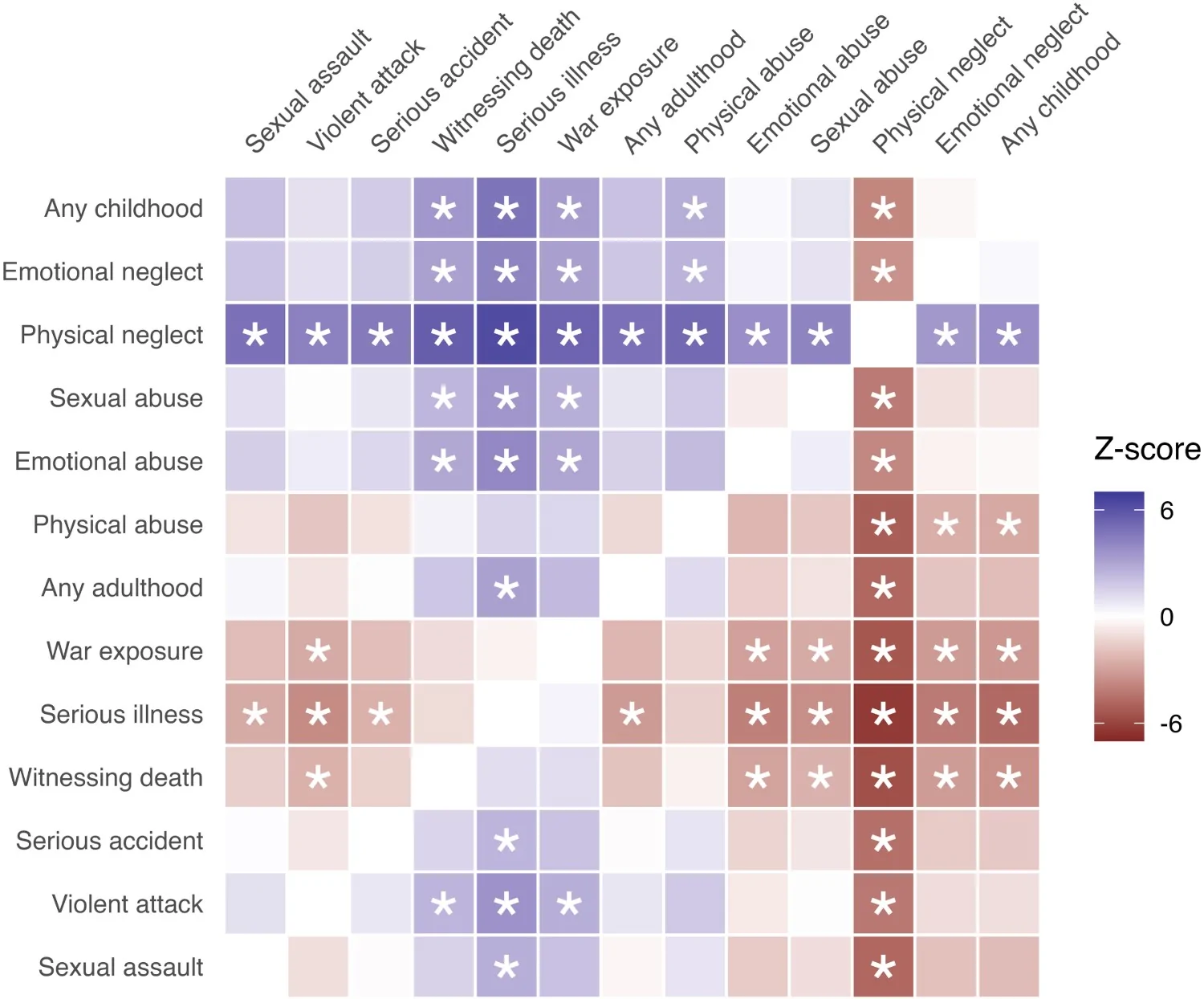
Z-score comparisons of PTSD PRS-trauma interaction effects between traumas. Note: PRS: Polygenic risk score. Results are based on the meta-analysed PRS-trauma interaction effects. A positive Z-score (blue tile) indicates the trauma on the *y*-axis had a greater interaction effect than the trauma on the *x*-axis. A negative Z-score (red tile) indicates the opposite. An asterisk * indicates a Z-score was statistically significant (78 tests, *q<*0.05). See Supplementary Table 6 for the Z-score values.

The PTSD PRS explained 2.6% of the PCL-6 score variance in GEC and 1.6% in UKB after controlling for genotyping batch and 10 genomic principal components. All meta-analysed PRS-trauma interactions were positive, as were all interactions in UKB and all but one (war exposure) in GEC (Figure 1; Supplementary Table 5). This meant that the association between traumas and PCL-6 scores almost always increased as genetic risk also increased. All PRS-trauma interactions except war exposure were statistically significant in the meta-analysis and UKB, while 6/13 were significant in GEC. Interactions were stronger on average among childhood traumas than adulthood traumas in both UKB (median β =   0.29 vs 0.19) and GEC (median β =   0.17 vs 0.10). Meta-analysed interactions differed significantly between traumas in 41% of Z-score comparisons, after false discovery rate (FDR) correction (78 tests, *q* < 0.05). Childhood physical neglect had a significantly greater meta-analysed interaction effect than all other traumatic experiences (Figure 2; Supplementary Table 6).

In our sensitivity analyses, the MDD PRS explained more of the variance in PCL-6 scores (3.3% in GEC, 2.2% in UKB) than the PTSD PRS, attributable to the greater statistical power of the MDD GWAS (Adams et al., 2025). The Z-score pattern was similar, indicating the results were consistent across correlated PRS, although childhood emotional neglect had a notably weaker interaction with the MDD PRS than the PTSD PRS (Supplementary Tables 7-8). When rerunning the PTSD PRS analyses with childhood traumas as ordinal variables (0-4), the Z-score pattern was highly similar (Supplementary Table 9), indicating variation in interactions was not due to the fact that some childhood traumas had stricter cutoffs than others. The estimates from the structural equation models used to assess the influence of gene-trauma correlations on interaction effects are reported in Supplementary Table 10. Freely estimating the PRS → trauma path resulted in attenuated interaction effects for most traumas (median 20% attenuation in GEC, 31% in UKB). Two out of 6 PRS-trauma interactions lost statistical significance in GEC (violent attack, physical abuse) and 2 out of 12 did in UKB (witnessing death, serious illness), meaning the majority of significant interactions remained.

Meta-analysed interaction effects had a moderate negative association with phi coefficient sums in UKB (*r* = −0.44) but not GEC (*r* = 0.10). This meant the more a trauma overlapped with others in UKB, the weaker its PRS interaction was. Interaction effects had small associations with PCL-6-trauma correlations (UKB: *r* = 0.16, GEC: *r* = 0.21), trauma exposure rates (UKB: *r* = −0.18, GEC: *r* = −0.03), and PRS-trauma correlations (UKB: *r* = 0.17, GEC: *r* = 0.13), indicating that variation in gene-environment interactions was not simply driven by these sources of trauma variation.

### 3.4. Gene-trauma interactions

In total, 124 of the 387 gene scores replicated in terms of a significant meta-analysed association with PCL-6 scores (387 tests, *q* < 0.05, Supplementary Table 11). Replication was greater in UKB (122 genes) than GEC (27 genes). Effect sizes were small, with individual gene scores explaining <0.1% of PCL-6 score variance in both cohorts. Replicating genes were most enriched for transcription factor binding (GO:0008134) compared to non-replicating genes, although this did not survive FDR correction (63 tests, *q* < 0.05; Supplementary Table 12). In GEC, 20 genes had at least one statistically significant interaction effect, compared to 6 in UKB (5,031 tests, *q* < 0.05; Supplementary Table 13). Ten genes had at least one significant meta-analysed interaction effect. This included two genes (*FAM120A, TMEM106B*) identified as potentially causal in the latest PGC PTSD GWAS (Nievergelt et al., 2024), although potentially causal genes were not significantly more likely than other genes to have significant meta-analysed interactions (Fisher’s exact *p* = .297). We found no significant enrichment comparing genes with and without significant interactions (Supplementary Table 14). Childhood traumas comprised 67% of significant interactions in UKB, 90% in GEC, and 82% of significant meta-analysed interactions.

**Figure 3.**
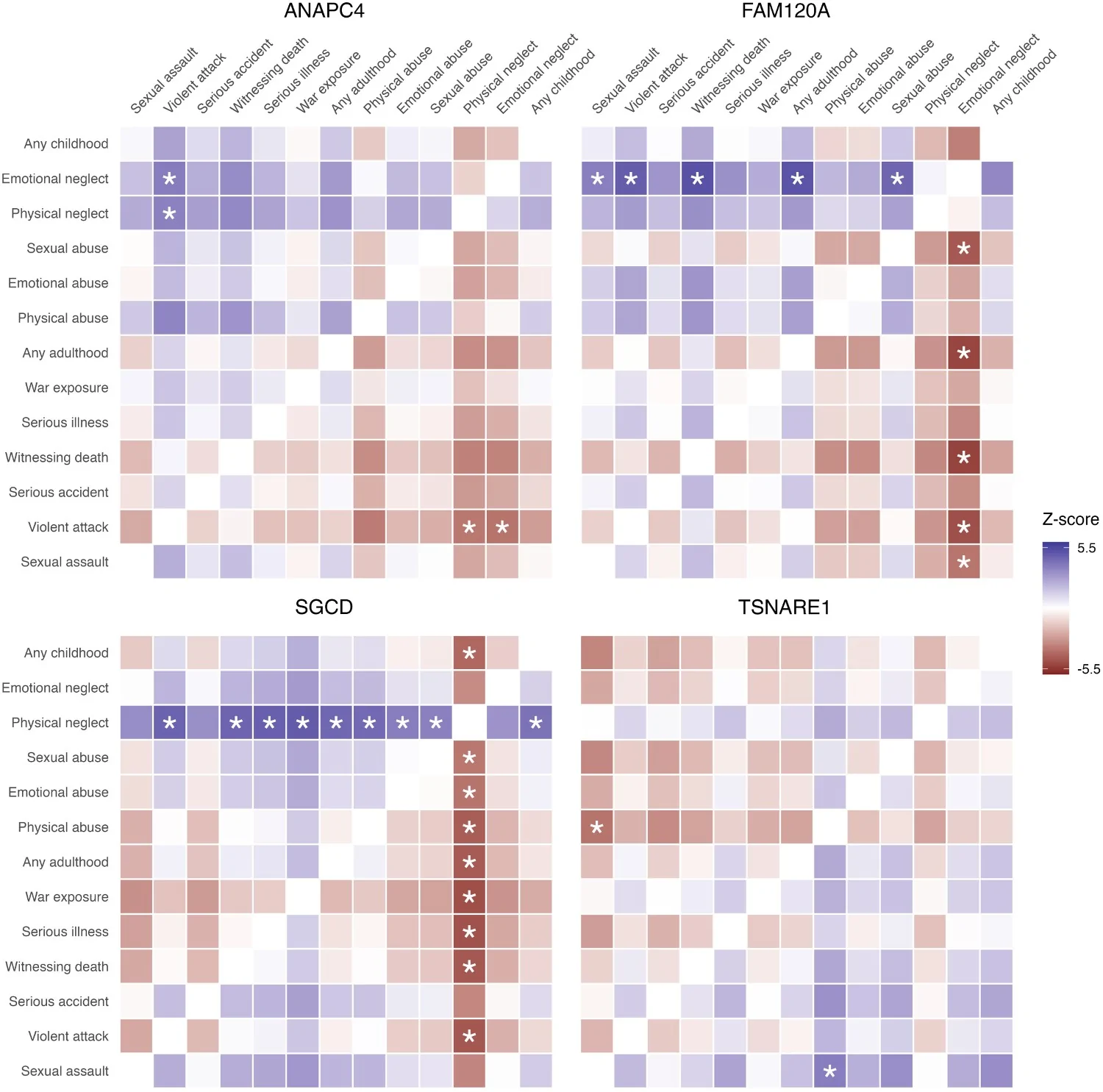
Examples of potentially causal genes from Nievergelt et al. (2024) with significant Z-score comparisons between traumas. Note: Results are based on the meta-analysed gene-trauma interaction effects. A positive Z-score (blue tile) indicates the trauma on the y-axis had a greater interaction effect than the trauma on the x-axis. A negative Z-score (red tile) indicates the opposite. An asterisk * indicates a Z-score was statistically significant (5,031 tests, *q* < 0.05). Other potentially causal genes with significant Z-scores included *GRI*A1, *IPO*11 and *TME*M106B.

**Figure 4.**
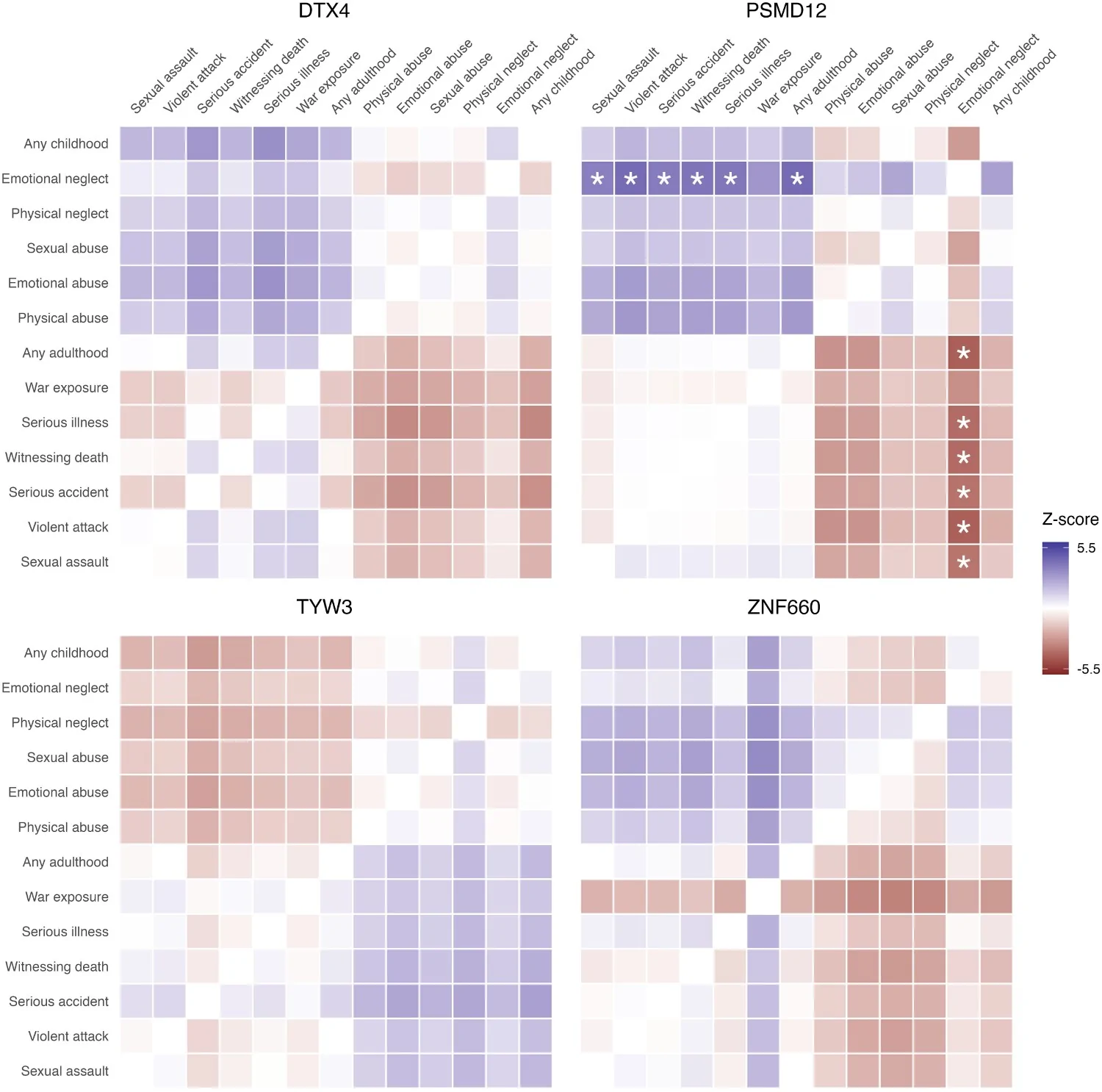
Genes with greater interaction effects for all childhood traumas or all adulthood traumas. Note: Results are based on the meta-analysed gene-trauma interaction effects. A positive Z-score (blue tile) indicates the trauma on the y-axis had a greater interaction effect than the trauma on the x-axis. A negative Z-score (red tile) indicates the opposite. An asterisk * indicates a Z-score was statistically significant (5,031 tests, *q* < 0.05). These genes all had statistically significant omnibus tests of childhood vs adulthood traumas, as did 17 other genes (Supplementary Table 14).

A total of 65 genes had significant differences in meta-analysed interactions between traumas (Z-score comparisons, 30,186 tests, *q* < 0.05), including seven potentially causal genes (*ANAPC4*, *FAM120A*, *GRIA1*, *IPO11*, *SGCD*, *TMEM106B*, *TSNARE1*; Figure 3). Potentially causal genes were not significantly more likely to have significant differences between traumas (Fisher’s exact *p* =   .592). Several genes interacted particularly strongly with a specific trauma, such as *SGCD* with childhood physical neglect. Four genes (*DTX4*, *PSMD12*, *TWY3*, *ZNF660*) had stronger meta-analysed interactions with either all childhood traumas or all adulthood traumas simultaneously (Figure 4). An additional 19 genes had a similar pattern of results with one trauma as an exception (e.g. *FAM120A* had stronger interactions with all childhood traumas except sexual abuse). Omnibus testing revealed significant differences between mean childhood trauma interactions and mean adulthood trauma interactions for 1 gene in GEC (*ANAPC4*), 17 genes in UKB, and 21 genes when meta-analysed (387 tests, *q* < 0.05; Supplementary Table 15). Genes with significant meta-analysed omnibus tests were most enriched in nervous system development (GO:0007399) and immune system process classes (GO:0001817/GO:0050852), although these did not survive FDR correction (Supplementary Table 16).

### 3.5. SNP-trauma interactions

Eight SNPs replicated in terms of a significant meta-analysed association with PCL-6 scores after controlling for genotyping batch and 10 genomic principal components (Supplementary Table 17). Replication was again greater in UKB (16 SNPs) than GEC (1 SNP), and individual SNPs explained <0.1% of PCL-6 score variance in both cohorts. Ten SNPs had at least one statistically significant interaction effect in UKB, while none did in GEC (1,235 tests, *q* < 0.05; Supplementary Table 18). Six SNPs had a statistically significant meta-analysed interaction effect. Childhood traumas comprised 80% of significant interactions in UKB and 83% of significant meta-analysed interactions. Interaction effects did not differ significantly by individual trauma for any SNPs in either cohort or when meta-analysed (Z-score comparisons, 7,410 tests, *q* < 0.05).

## 4. Discussion

We aimed to quantify the extent to which gene-environment interactions in PTSD differ among specific traumas. Through comprehensive analyses incorporating polygenic risk scores, genes, and variants, we found that traumas differed notably in the magnitude of their gene-environment interactions. We found that PTSD PRS interacted more strongly with childhood traumas than adulthood traumas on average, and particularly childhood neglect. We found that some potentially causal genes implicated by GWAS interacted particularly strongly with certain traumas. For example, *FAM120A* interacted significantly more strongly with childhood emotional neglect than all other traumas. Other potentially causal genes, including *ANAPC4*, *GRIA1*, *IPO11*, *TMEM106B*, *SGCD*, and *TSNARE1*, interacted most strongly with a broad range of other traumas, most often during childhood. We identified 21 genes that interacted significantly more strongly with either childhood or adulthood traumas, suggesting the effects of certain genes might depend on developmental period of exposure. Overall, our findings are consistent with differential gene-environment interactions across specific traumas and highlight the complex interplay between specific genes, specific traumas, and PTSD.

Our results suggest that some gene-wise effects in PTSD previously identified in GWAS may be moderated by specific traumas. We identified 10 genes that had significant interaction effects with specific traumas. This included two potentially causal genes: *FAM120A* with emotional neglect and *TMEM106B* with physical neglect. An additional 55 genes had no significant interaction effects but did have significant differences in interaction effects between traumas, providing additional evidence for trauma-specific interactions. Similarly, 6 of the 95 lead SNPs for PTSD had significant interaction effects with specific traumas. Of note, *rs13237518* and its associated gene *TMEM106B* both interacted significantly with childhood physical neglect, strengthening evidence for this interaction. Our findings suggest that individual genes can associate more strongly with PTSD in the presence of certain traumas. For example, certain variation in a gene such as *FAM120A* might contribute more to PTSD risk following childhood emotional neglect than other traumas. We conducted enrichment analyses comparing genes with and without significant interaction effects, although this was restricted to 63 gene ontology classes based on the number of genes we examined. Future research to further explore variation among traumas at the pathway level would facilitate better understanding of why a given gene might interact strongly with a given trauma.

Our findings highlight an apparent distinction between childhood and adulthood traumas in their relation to genetic risk and PTSD. We found that childhood traumas had stronger associations with PTSD symptoms on average. This is despite adulthood traumas more closely matching the DSM-5/ICD-11 descriptions of trauma. Childhood traumas have long been found to associate with mental health problems such as PTSD in adulthood (Jaffee, 2017; Kessler et al., 2010). Our results are consistent with previous findings that childhood traumas can be more strongly associated with mental health problems in adulthood than adulthood traumas (Dunn et al., 2017; Zlotnick et al., 2008). Polygenic risk scores for PTSD interacted more strongly with childhood traumas on average, as did most of the specific genes examined. This aligns with previous findings that PTSD PRS interactions are stronger with childhood traumas (Bugiga et al., 2024; Huckins et al., 2020), and is consistent with the possibility that genetics plays a more important role in shaping how people respond to childhood traumas than adulthood traumas.

One potential explanation for our stronger observed gene-environment interactions with childhood traumas is that they occur in a more critical period of biological and psychological development (Cross et al., 2017) and therefore reflect qualitatively distinct processes. We found that genes with stronger childhood trauma interactions were particularly enriched for pathways related to nervous system development and the immune system, which has been proposed as a mediator between childhood trauma and psychopathology in later life (Danese & Lewis, 2017). However, this enrichment was only statistically significant before and not after FDR correction, and so caution is warranted when interpreting this finding. Alternative explanations might be that differences between childhood and adulthood traumas reflect certain methodological factors, such as the accuracy of retrospective self-reports, or epidemiological factors, such as trauma co-occurrence, cumulative exposure, or the influence of traumas on mental and physical health beyond PTSD symptoms. We found that traumas which were more intercorrelated did not have stronger interactions, which suggests that interactions were not simply inflated by the tendency of a trauma to co-occur with others. We also found little association between gene-environment interactions and gene-environment correlations among traumas, and that the majority of significant interactions remained even when explicitly modelling gene-environment correlation. This similarly suggests that interactions were not simply explained by gene-environment correlations, despite varying across traumas. However, other explanations could not be further tested using the data we analysed in this study, which makes it challenging to assess whether differences between childhood and adulthood traumas truly reflect qualitatively distinct interaction processes.

We also observed notable differences between childhood abuse and childhood neglect. Abuse was more strongly associated with PTSD symptoms, while neglect had stronger gene-environment interactions. In other words, the influence of childhood abuse on PTSD was stronger and more consistent, while the influence of childhood neglect was more moderate and contextual. Genetics might be particularly important in shaping how people respond to childhood neglect, beyond both childhood abuse and adulthood traumas. Some research has suggested that childhood abuse and neglect have distinct effects on brain development (McLaughlin et al., 2014), which might help explain differences in gene-environment interactions, although this is disputed (Nelson & Gabard-Durnam, 2020). It is also possible that the observed differences in gene-environment interactions between abuse and neglect again reflect differences in underlying factors such as self-report accuracy or cumulative exposure, which could not be tested here. Childhood emotional and physical neglect can have severe effects on mental and physical health (Lippard & Nemeroff, 2020; Nelson et al., 2023) and are common among adults with PTSD (Carvalho Silva et al., 2024). However, this has often been overlooked in research (Carvalho Silva et al., 2024), and emotional and physical neglect are not listed as example traumas in the DSM-5 or ICD-11, influencing clinical practice. Our finding that childhood emotional and physical neglect associated with adulthood PTSD symptoms and had stronger interaction effects with genetic risk than other traumas suggests that they have an important place in PTSD research. More broadly, it highlights that valuable information might be gained about PTSD when a wider range of potential traumas are accommodated.

Trauma exposure should not be neglected in future genomic research on PTSD. Incorporating trauma data has been found to improve detection of genetic variants associated with PTSD (Maihofer et al., 2022). Unless only trauma exposed participants are included in gene association analyses, it is difficult to assess whether genetic variants are associated with risk for PTSD following trauma or with trauma self-reporting (Peel et al., 2022). Our finding that traumas can differ quantitatively from one another in their gene-environment interactions presents a challenge for generalising gene associations across populations with different trauma exposures. If, for example, a gene interacts with childhood traumas more strongly than adulthood traumas (as was the case for many genes in this study), it is likely that the degree of association between that gene and PTSD will differ between populations with different rates of childhood trauma exposure. There is some evidence to support this. A recent study found an association between *FAM120A* and PTSD in female but not male participants (Moo-Choy et al., 2025), which is compatible with our findings that *FAM120A* interacted more with childhood traumas than adulthood traumas and that childhood traumas were more common in female participants. Given the potentially moderating effects of trauma type on gene associations, researchers should aim to account for trauma exposure whenever feasible. This might improve detection of genetic signal unique to people with certain trauma exposures and further our understanding of the complex interplay between specific genetic factors, specific environmental factors, and PTSD.

It is important to consider the extent to which our results replicate those of the latest PGC GWAS of PTSD (Nievergelt et al., 2024), as this was the base for our polygenic risk scores and implicated the variants and genes we examined. In total, 124 of the 387 genes we examined replicated in terms of a significant meta-analysed association with PTSD symptom scores, including 18 of the 43 potentially causal genes (e.g. *CACNA1E*, *DCC*, *GRIA1*). However, only 8 of the 95 lead genome-wide significant SNPs replicated in terms of a significant meta-analysed association with PTSD symptom scores. This is despite the fact that these SNPs were selected from a GWAS that included the UK Biobank, which should inflate associations within this cohort. Still, 62 of the 95 SNPs had a consistent direction of effect between our study and the PGC GWAS, beyond the level expected under a null binomial model (*p* =   .002). This suggests that we largely captured the same effects as the GWAS, but at a lower precision (and therefore non-significance) due to our restricted sample size.

There were several limitations of this study. We examined individual genes and genetic variants with significant main effects reported in previous genome-wide research (Nievergelt et al., 2024). This was not a genome-wide gene-environment interaction study, and our approach cannot identify novel interactions between trauma exposure and variants without significant main effects on PTSD from GWAS. Future genome-wide interaction studies are warranted to provide a more comprehensive exploration of gene-trauma interactions in PTSD. Genome-wide interaction studies of depression (Arnau-Soler et al., 2019; Dunn et al., 2016; Ikeda et al., 2016) and suicidality (Wendt et al., 2021) have shown this to be a valuable approach. However, these studies require extremely large sample sizes for adequate power, exceeding those of GWAS (Herrera-Luis et al., 2024).

Our cohorts were restricted to participants recruited in the United Kingdom, which limits the generalisability of the results. Patterns of gene-environment interactions can vary across countries and communities based on environmental differences (e.g. differences in trauma exposure/reporting) and differences in genetic ancestry (Herrera-Luis et al., 2024), and so greater diversity in gene-environment interaction research moving forward is needed. Our cohorts were also notably heterogeneous. The UK Biobank represents an older, population-based sample, while the GLAD-EDGI-COPING studies represent a younger and more sex-imbalanced sample specifically recruited based on lived experience of mental health problems. This was reflected in differences in both self-reported trauma exposure and PTSD symptoms between the two cohorts. Gene-environment interactions estimated in clinically enriched samples may partly reflect different underlying mechanisms than those observed in population-based samples, such as differences in baseline risk, self-report, and treatment seeking/response. The reporting of cohort-specific results was therefore emphasised, with meta-analysed results reported additionally. We used fixed-effect meta-analysis because in the case of two heterogeneous cohorts, statistical tests of heterogeneity and random-effects models have limited efficacy (Friede et al., 2017). Gene-environment interactions in samples with clinically diagnosed PTSD might similarly reflect differences in underlying mechanisms (e.g. self vs clinician report, treatment seeking/response), and so future research comparing interactions in clinical and non-clinical samples would be valuable.

Based on the data available in our cohorts, we incorporated eleven trauma exposures in this study, but this was not an exhaustive set. Future research might investigate gene-environment interactions in other traumas, such as those included in the WHO World Mental Health Surveys (Kessler et al., 2017) but not included here (e.g. natural disasters, stalking). Particularly valuable would be studies of gene-environment interactions in potentially traumatic experiences that have historically been neglected, such as difficult childbirth (Ahsan et al., 2023), mental health crises (Berry et al., 2013), or racial trauma (Williams et al., 2021). The trauma exposure measures in this study were self-reports, which studies have shown can misalign with objective and prospective reports of trauma exposure (Baldwin et al., 2019). Future research might investigate gene-environment interactions with objective/prospective trauma exposures. The measures were also limited in detail, restricted to one item per trauma and, for adulthood traumas, restricted to binary yes/no responses. This is common in Biobank-scale studies, which are broadly phenotyped to maximise use by the research community (Coleman, 2021). These studies are useful for genomic research, which requires very large sample sizes to assess small effects with the necessary precision, and which is more effective within large samples with consistent measurement than many smaller samples meta-analysed (Assary et al., 2018). This also meant, however, that highly heterogeneous traumatic experiences were aggregated into broad totalising categories, and important dimensions of trauma exposure such as timing, chronicity, and context could not be assessed. Insofar as traumatic experiences share these dimensions across categorical bounds, comparing broad trauma categories (e.g. abuse vs neglect) risks imprecision and explaining differences in findings among categories becomes challenging. Currently, no trauma exposure measure exists that successfully captures all potentially trauma experiences while also assessing context such as timing, frequency, and duration, and so this challenge is pervasive throughout the literature (Karstoft & Armour, 2023). Dimensions like these are relevant for understanding gene-environment interaction in PTSD mechanistically, and so future research aimed at investigating gene-environment interactions in relation to these is needed. To give an example, although we found important differences between childhood and adulthood traumas, we did not have data to examine different periods of childhood. Studies have produced conflicting evidence about whether certain developmental periods are most sensitive to the effects of trauma (Dunn et al., 2016). If childhood is a critical period for the interplay between trauma and genetic risk for PTSD, as our study suggests, future research should aim to further investigate this interplay in different developmental periods.

Our findings demonstrate that the genetic architecture of PTSD cannot be fully understood without consideration of the specific potentially traumatic experiences individuals have. By comparing eleven traumas across two large cohorts and integrating analyses of polygenic risk scores, genes, and SNPs, we showed that gene-environment interactions in PTSD can vary in magnitude across potentially traumatic experiences and are especially pronounced for childhood emotional and physical neglect. Our results underscore the need for future genomic research to incorporate trauma exposure data and highlight the value of moving beyond trauma-general models. A more nuanced understanding of how particular traumas interact with genetic factors has the potential to refine aetiological models of PTSD, improve the interpretability of genetic findings across diverse populations, and ultimately inform more targeted approaches to prevention and intervention.

## Supplementary Material

EJPT_revised_supp_method_2_20260716.docx

EJPT_revised_supp_tables_anon_20260529.xlsx

## Data Availability

GLAD-EDGI-COPING study data are available via data request application (https://bioresource.nihr.ac.uk/using-our-bioresource/academic-and-clinical-researchers/apply-for-bioresource-data/). These data are not publicly available due to restrictions outlined in the study protocol and specified to participants during the consent process. Deidentified data from UK Biobank is available to bona fide researchers submitting an approved project application. Bespoke code underlying the primary analyses in this paper will be made available on publication at https://github.com/ColemanResearchGroup.
