# Supplementary material for: Identifying differential interactions of potentially traumatic experiences with genetic risk for posttraumatic stress disorder
Source: Eur J Psychotraumatol. 2026 Sep 16;17(1):2709319. doi: 10.1080/20008066.2026.2709319 (PMC13587574; doi:10.1080/20008066.2026.2709319)
Supplement: Supplementary file 1 [file ZEPT_A_2709319_SM5879.docx]

# Supplementary Method

## 1. Cohort genotyping, imputation and QC

|  | **GLAD-EDGI-COPING** | **UK Biobank** |
| --- | --- | --- |
| Genotyping Array | UK Biobank Axiom | UK Biobank Axiom |
| Subject level QC |  |  |
| Subject call rate | > 0.95 | > 0.98 |
| Sex check | F_male_ < 0.8, F_female_ > 0.5 | F_male_ < 0.9, F_female_ > 0.5 |
| Heterozygosity | F > -0.2, F < 0.2 | See note |
| Genotype level QC |  |  |
| Genotyping call rate | > 0.95 | > 0.98 |
| MAF | > 0.01 | > 0.01 |
| HWE | *p* < 1e-10 | *p* < 1e-8 |
| Imputation |  |  |
| Phasing software | EAGLE2 | SHAPEIT3 |
| Imputation software | Minimac4 | IMPUTE4 |
| Imputation panel | TOPMed r2 | HRC + 1000G P3 |
| Post-imputation QC |  |  |
| INFO | > 0.8 | > 0.8 |
| MAF | > 0.01 | > 0.01 |
| Sample exclusions |  |  |
| Genetic relatedness | KING < 0.0084 | KING < 0.0084 (via ukbtools *R* package) |
| Genetic ancestries | EUR only | EUR only (field 30079) |

*Note*. For information regarding heterozygosity outliers in the UK Biobank, see <https://doi.org/10.1038/s41586-018-0579-z>.

## 2. PRScs parameters

These were the same for GLAD-EDGI-COPING and UK Biobank. The --phi parameter was not manually specified and was therefore learned from the data.

--a=1

--b=0.5

--n_iter=1000

--n_burnin=500

--thin=5

--beta_std=False

--write_psi=False

--write_pst=False

## 3. PRSet parameters

These were the same for GLAD-EDGI-COPING and UK Biobank.

--clump-kb 1000kb

--clump-p 1

--clump-r2 0.3

--full-back

--proxy 0.8

--set-perm 10000

## 4. PCL-6 items with response options and UKB field IDs

*Next is a list of problems and complaints that people sometimes have in response to such extremely stressful experiences. Please indicate how much you have been bothered by that problem in the past month:*

|  | Not at all | A little bit | Moderately | Quite a bit | Extremely |
| --- | --- | --- | --- | --- | --- |
| Repeated, disturbing memories, thoughts, or images of a stressful experience [UKB 20497] |  |  |  |  |  |
| Feeling very upset when something reminded you of a stressful experience [UKB 20498] |  |  |  |  |  |
| Avoiding activities or situations because they reminded you of a stressful experience [UKB 20495] |  |  |  |  |  |
| Feeling distant or cut off from other people [UKB 20496]* |  |  |  |  |  |
| Feeling irritable or having angry outbursts [UKB 20494]* |  |  |  |  |  |
| **GLAD-EDGI-COPING only**  Difficulty concentrating |  |  |  |  |  |
| **UK Biobank only**  Trouble concentrating on things, such as reading the newspaper or watching television [UKB 20508]** |  |  |  |  |  |

*Note*. An asterisk * indicates an item was only shown in the UK Biobank if participants responded “a little bit” or greater to any of the first three items. ** The response options for this item were “not at all”, “several days”, “more than half the days”, and “nearly every day”.

## 5. Childhood Trauma Screener items with exposure cutoffs and UKB field IDs

*When I was growing up…*

|  | Never | Rarely | Sometimes | Often | Very Often |
| --- | --- | --- | --- | --- | --- |
| **Emotional neglect**  I felt loved [UKB 20489] | Exposed | |  |  |  |
| **Physical abuse**  People in my family hit me so hard that it left me with bruises or marks [UKB 20488] |  |  | Exposed | | |
| **Emotional abuse**  I felt that someone in my family hated me [UKB 20487] |  |  | Exposed | | |
| **Sexual abuse**  Someone molested me (sexually) [UKB 20490] |  | Exposed | | | |
| **Physical neglect**  There was someone to take me to the doctor if I needed it [UKB 20491] | Exposed | |  |  |  |

*Note*. Participants were flagged as trauma exposed if their response to a given item fell within the ranges displayed above. For example, a participant was exposed to emotional abuse if their responses to that item were “sometimes”, “often”, or “very often”.

## 6. Protein coding genes for which PRSet did not produce risk scores

AP001350.1

CNKSR2

HIST1H2AE

HIST1H2AI

HIST1H2AK

HIST1H2AM

HIST1H2BH

HIST1H2BI

HIST1H2BM

HIST1H2BO

HIST1H3E

HIST1H3F

HIST1H4D

HIST1H4K

HIST1H4L

IGSF1

LRRC37A

LRRC37A2

MAMLD1

MTM1

MTMR1

NDUFAF3

OR5B21

PCYT1B

POLA1

UCN

*Note.* These 28 genes were among the 415 taken from Supplementary Table 9 of the 2024 PGC PTSD GWAS paper. The remaining 387 genes were included in the analyses.

## 7. Sensitivity analyses of PRS-trauma interaction effects controlling for PRS-trauma correlations

We used structural equation modelling to re-estimate our PRS–trauma interaction effects on PTSD symptoms while explicitly modelling the association between the PRS and trauma. This would allow us to assess the extent to which interactions attenuated after statistically controlling for gene-environment correlation. For each trauma, we compared two structural equation models: one in which a PRS → trauma path was freely estimated and one in which it was constrained to zero (which corresponds to the primary analyses). All covariates from the primary analyses were retained. An illustrative figure of these two models is presented below. We ran models using the “lavaan” *R* package, with WLSMV estimation to accommodate variables of mixed type (ordinal, continuous). The results of these analyses for each cohort, including the PRS-trauma interactions from both models and the freely estimated PRS-trauma paths, are reported in Supplementary Table 10. All models had acceptable fit indices (RMSEA < 0.08, CFI > 0.9, TLI > 0.9).

*Note.* PRS = Polygenic risk score. For the purpose of simplicity, a single covariate is included for reference rather than all covariates. Paths from a variable to another path indicate moderation/interaction effects (e.g. PRS-trauma interaction) estimated via product terms.
